# Mitral and Aortic Regurgitation in Patients Undergoing Kidney Transplantation: The Natural Course and Factors Associated With Progression

**DOI:** 10.3389/fcvm.2022.809707

**Published:** 2022-01-27

**Authors:** Minjeong Kim, Darae Kim, Juhan Lee, Dae-Young Kim, Jiwon Seo, Iksung Cho, Kyu Ha Huh, Geu-Ru Hong, Jong-Won Ha, Chi Young Shim

**Affiliations:** ^1^Division of Cardiology, Severance Cardiovascular Hospital, Yonsei University College of Medicine, Seoul, South Korea; ^2^Division of Cardiology, Department of Medicine, Heart Vascular Stroke Institute, Samsung Medical Center, Sungkyunkwan University School of Medicine, Seoul, South Korea; ^3^Departement of Surgery, Yonsei University College of Medicine, Seoul, South Korea

**Keywords:** left-side valve disease, mitral regurgitation, aortic regurgitation, ESRD, kidney transplantation

## Abstract

**Background:**

Valve regurgitation can decrease with resolution of hemodynamic loads on the left ventricle (LV) after kidney transplantation (KT). We aimed to investigate the natural course of left-side valve regurgitation after KT and factors associated with progression.

**Methods:**

Among patients who underwent KT in two tertiary centers, 430 (224 men, mean age 50 ± 13 years) were examined by echocardiography within 3 months before KT and between 6 and 36 months after KT. Mitral regurgitation (MR) and aortic regurgitation (AR) were graded according to the current guidelines. Regression was defined as a decrease in regurgitation by one or more steps, and progression was an increase in one or more steps after KT. Clinical and echocardiographic factors associated with progression of MR and AR were analyzed.

**Results:**

Mild or greater MR was observed in 216 (50%) patients before KT, and mild or greater AR was observed in 99 (23%). During the follow-up period of 23.4 ± 9.9 months, most patients experienced regression or no change in regurgitation after KT, but 34 patients (7.9%) showed MR progression and 37 (8.6%) revealed AR progression. Patients who showed MR progression were more likely to receive a second KT, have mitral annular calcifications, and show a smaller decrease in LV end-systolic dimension. Patients who showed AR progression were more likely to have persistent hypertension after KT, aortic valve calcifications, and a smaller reduction of LV end-systolic dimension.

**Conclusions:**

Risk factors for progression of MR after KT include a second KT, MAC and a smaller decrease in LV end-systolic dimension after KT. Risk factors for progression of AR include valve calcification, persistent hypertension and a smaller decrease in LV end-systolic dimension after KT. Further echocardiographic surveillance and risk factor management after KT are warranted in these patients.

## Introduction

Valve regurgitation is observed frequently in patients with chronic kidney disease or end stage renal disease (ESRD) (1, 2). Left-side valve regurgitation, including mitral regurgitation (MR) and aortic regurgitation (AR), is predicted to decrease when the hemodynamic load on the left ventricle (LV) decreases after kidney transplantation (KT). However, in some patients, left-side valve regurgitation does not decrease but persists or even progresses (3–5). In particular, degeneration and structural change of the valve start early in patients with ESRD because of the hemodynamic load and impaired calcium-phosphate homeostasis and progress faster than in those with normal kidney function (6–8). MR and AR are affected by different hemodynamic factors and structural alterations. MR is influenced more highly by preload and LV remodeling, while AR is affected most by afterload and aorta remodeling. Patients who have undergone KT are thought to have reduced or increased MR or AR depending on the clinical situation in pre-KT and post-KT periods (9). However, data regarding the prevalence of left-side valve regurgitation in patients undergoing KT and the natural course of MR and AR after KT are scarce.

In the present study, we investigated the prevalence of MR and AR before KT and the regression or progression rate of MR and AR after KT. We also identified clinical and echocardiographic factors associated with progression of MR and AR.

## Methods

### Study Population

This study included patients with ESRD who received KT between 2005 and 2018 at two tertiary medical centers (Severance Hospital, Seoul, Korea; Samsung Medical Center, Seoul, Korea). Patients with a history of valve surgery or intervention, congenital heart disease, or combined transplantation with other organs were excluded, as were patients without follow-up transthoracic echocardiography (TTE) after KT. After applying exclusion criteria, 430 patients were analyzed retrospectively.

All patients underwent TTE at least twice. Pre-KT echocardiography was performed within 3 months before KT, and post-KT echocardiography was performed between 6 and 36 months after KT. If a patient underwent TTE more than twice after KT, we analyzed the most recent.

Clinical information was obtained from electronic medical records, and data were analyzed at baseline and follow-up TTE. Prior medical history was composed of hypertension, diabetes mellitus, dyslipidemia, atrial fibrillation, coronary artery disease, heart failure, duration of hemodialysis, and second KT. The diagnosis of heart failure was based on typical clinical symptoms and signs caused by a structural or functional cardiac abnormality, followed the current guideline, including both reduced EF and preserved EF (8). Systolic and diastolic blood pressures were measured at baseline and post-KT follow-up visits. Clinical factors after KT including new-onset hypertension, new-onset diabetes, renal dysfunction, graft failure, and cardiovascular medications were collected. Renal dysfunction was defined as a >1.5-fold increase in serum creatinine or an absolute increase in serum creatinine ≥ 0.3 mg/dL (9).

### Echocardiography

Two-dimensional and Doppler echocardiography was performed using a commercially available ultrasound machine with a 2.5–3.5 MHz probe before and after KT. Standard measurements were performed following the current guideline recommendations (10). LV ejection fraction (EF) was measured using the biplane Simpson's method in apical four- and two-chamber views. Left atrial (LA) volume index was measured by the biplane method at the end of ventricular systole and indexed to body surface area. From the mitral inflow velocities, we obtained data on peak velocity of early (E) and late filling and deceleration time of E velocity. Early diastolic (e′) velocities were measured at the septal mitral annulus (11).

The severity and mechanism of each valve regurgitation were assessed according to American Society of Echocardiography guidelines (12). The degrees of MR and AR, if present, were graded as no/trivial, mild, moderate, or severe using an integrated approach (12). The etiology of MR was categorized into primary or secondary MR. Primary MR was defined as degenerative MR directly affecting the mitral valve leaflets and/or chordae, and secondary MR was MR due to a pathological process of the LV or LA (12). Mitral annular calcification (MAC) was defined as the thick and echo-dense area of the mitral annulus, occasionally extending to mitral valve leaflets, as described in previous studies (13, 14). Aortic valve calcification was defined as a calcium deposit in the aortic root and valve regardless of restriction of leaflet mobility on parasternal short- and long-axis views (15, 16). The echocardiography reports were independently reviewed by 2 cardiologists blinded to the clinical data. All discrepancy of echocardiographic readings was resolved by consensus. In a consensus process, the 2 cardiologists reviewed the echocardiographic images and reached an agreement on the interpretation.

Regression of MR or AR was noted if severity decreased by one or more grades. Progression was defined as severity increase by one or more grades. Patients were categorized into three groups (regression, unchanged, progression) according to valve regurgitation.

### Statistical Analysis

The baseline characteristics are expressed using frequencies and percentages for categorical variables using chi-square test. The continuous variables are summarized as mean ± standard deviation and were compared using analysis of variance (ANOVA) among the three groups. For pairwise comparisons, *post-hoc* test with Tukey's HSD was conducted. To identify factors associated with progression of valve regurgitation, a linear regression model was used. Univariable factors with *P* < 0.10 or the major relevant clinical factors were entered into multivariable analyses. The coefficient values were generated, and all two-sided *p*-values < 0.05 were considered statistically significant. The Hosmer–Lemeshow test for stepwise logistic regression was performed for incremental value of risk prediction. All statistical analyses were performed using SPSS software (version 25.0; IBM Corp., Armonk, NY, USA).

## Results

### Severity and Changes of Left-Side Valve Regurgitation After KT

**Figure 1:**
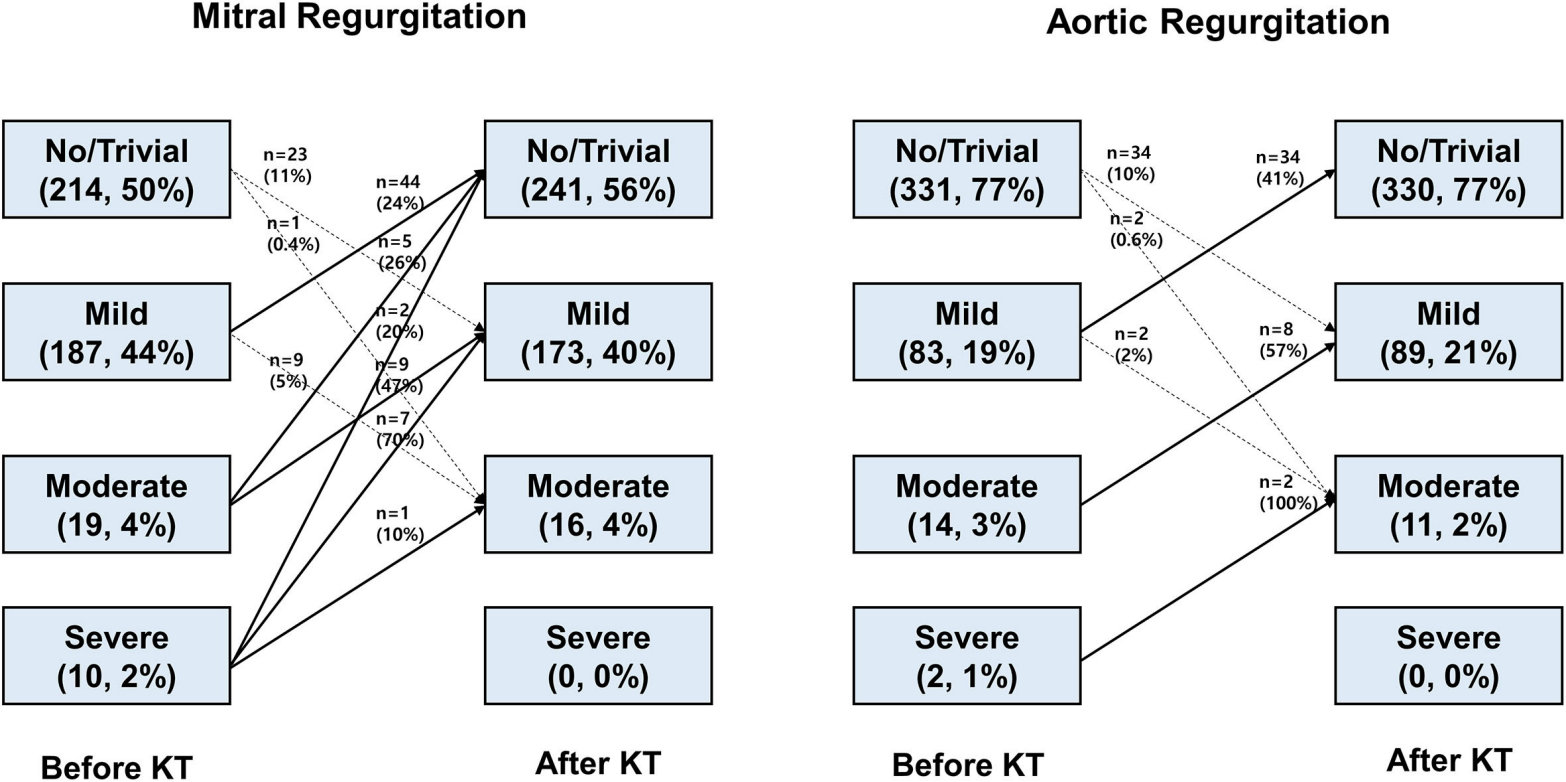
Natural course of left-sided valvular regurgitation after kidney transplantation.

The severity of MR and AR before KT and the changes after KT are shown in Figure 1. Before KT, 187 (44%) patients had mild MR, 19 (4%) had moderate MR, and 10 (2%) showed severe MR. Factors related to MR severity before KT are presented in Supplementary Table 1. Female sex, history of heart failure, and larger LA volume index were associated with MR before KT. Before KT, 83 (19%) patients had mild AR, 14 (3%) had moderate AR, and 2 (1%) showed severe AR. Factors associated with AR severity before KT are described in Supplementary Table 2. Old age and history of heart failure were associated with AR grade before KT. Mild or greater MR was observed in 216 (50%) patients and mild or greater AR in 99 (23%) before KT. During the follow-up period of 23.4 ± 9.9 months, most patients experienced regression or no change in regurgitation after KT, but 34 (7.9%) showed MR progression and 37 (8.6%) revealed AR progression.

### Factors Associated With MR Progression

Table 1 shows the baseline clinical and renal characteristics in the three groups according to change in MR after KT. A history of atrial fibrillation, history of heart failure and secondary MR tended to be more prevalent in the MR regression group compared with the other groups. Second KT and post-KT renal dysfunction were significantly different in the three groups, and these events tended to be more prevalent in the progression group. Most of the echocardiographic findings differed between the three groups. In patients with MR progression, LV chamber size was small, and E/e′ was lower than that in the other groups before KT, but these factors were significantly higher after KT. In the group showing MR progression, LV and LA size increased after KT, LVEF decreased, and E/e′ increased (Table 2).

**Table 1:** Comparison of clinical and renal characteristics in the three groups according to change in mitral regurgitation.

	**MR regression** **(***n*** = 68)**	**MR unchanged** **(***n*** = 328)**	**MR progression** **(***n*** = 34)**	***p*** **-value**
**Clinical characteristics**				
Age, years	51.0 ± 11.6	49.5 ± 13.0	53.9 ± 10.7	0.128
Male sex, *n* (%)	28 (40.6)	180 (55.0)	16 (47.1)	0.076
Hypertension, *n* (%)	58 (84.1)	299 (91.7)	29 (85.3)	1.000
Diabetes mellitus, *n* (%)	24 (34.8)	114 (35.0)	16 (47.1)	0.368
Dyslipidemia, *n* (%)	16 (23.2)	62 (19.0)	3 (8.8)	0.214
Atrial fibrillation, *n* (%)	9 (13.0)	13 (4.0)^*^	0 (0.0)^#^	0.003
CAD, *n* (%)	9 (13.0)	37 (11.3)	7 (20.6)	0.292
Heart failure, *n* (%)	13 (18.8)	24 (7.3)^*^	2 (5.9)^#^	0.008
**Renal characteristics**				
Dialysis, *n* (%)	68 (100.0)	328 (100.0)	34 (100.0)	1.000
Hemodialysis, *n* (%)	62 (91.2)	282 (86.0)	31 (91.2)	0.664
Peritoneal dialysis, *n* (%)	6 (8.8)	46 (14.0)	3 (8.8)	0.306
HD duration, months	56.7 ± 58.2	65.2 ± 66.9	56.4 ± 62.1	0.577
Second KT, *n* (%)	2 (2.9)	23 (7.0)	7 (20.6)^*^^#^	0.005
**Post-KT comorbidities**, ***n*** **(%)**				
New-onset HTN	4 (5.8)	13 (4.0)	2 (5.9)	0.732
Persistent HTN	13 (18.8)	103 (31.5)^*^	17 (50.0)^*^^#^	0.005
New-onset DM	5 (7.2)	52 (16.0)	3 (8.8)	0.111
Renal dysfunction, *n* (%)	9 (13.0)	62 (19.0)	12 (35.3)^*^^#^	0.025
Graft failure, *n* (%)	11 (15.9)	33 (10.1)	6 (17.6)	0.202
**Post-KT medications**				
RAAS blocker, *n* (%)	12 (18.8)	74 (26.9)	5 (15.6)	0.186
Beta blocker, *n* (%)	23 (34.8)	145 (52.3)^*^	14 (43.8)	0.032
CCB, *n* (%)	25 (36.0)	108 (32.9)	17 (50.0)	0.470
Diuretics, *n* (%)	6 (9.4)	27 (9.9)	4 (12.1)	0.906
Statin, *n* (%)	17 (26.2)	85 (31.4)	12 (36.4)	0.556

* *P < 0.05 compared with the MR regression group*.

# *P < 0.05 compared with the MR unchanged group*.

*MR, mitral regurgitation; CAD, coronary artery disease; HD, hemodialysis; KT, kidney transplantation; HTN, hypertension; DM, diabetes mellitus; RAAS, renin-angiotensin-aldosterone system; CCB, calcium channel blocker*.

**Table 2:** Comparison of echocardiographic characteristics in the three groups according to change in mitral regurgitation.

	**MR regression** **(***n*** = 68)**	**MR unchanged** **(***n*** = 328)**	**MR progression** **(***n*** = 34)**	***p*** **-value**
**Pre-KT echocardiogram**				
LVEDD, mm	56.6 ± 6.9	52.4 ± 5.9	51.3 ± 5.7^*^	<0.001
LVESD, mm	38.5 ± 9.3	34.1 ± 6.0	62.5 ± 6.1^*^	<0.001
LVEF, %	56.7 ± 12.8	62.3 ± 9.3	61.7 ± 10.6	<0.001
LV mass index, g/m^2^	148.4 ± 51.1	124.9 ± 37.0	118.6 ± 36.4	<0.001
LA volume index, ml/m^2^	51.6 ± 24.5	38.1 ± 15.1	39.5 ± 14.8	<0.001
E/e′	15.4 ± 6.9	12.4 ± 5.6^*^	12.2 ± 4.1^*^	0.001
PASP, mmHg	40.0 ± 11.8	30.3 ± 9.3^*^	35.4 ± 10.5	0.015
Degree of MR, *n* (%)				<0.001
No/trivial	0 (0)	190 (57.8)^*^	24 (70.6)^*^^#^	
Mild	46 (66.7)	131 (40.1)^*^	10 (29.4)^*^	
Moderate	12 (17.4)	7 (2.1)^*^	0 (0.0)^*^	
Severe	10 (14.5)	0 (0.0)^*^	0 (0.0)^*^	
Secondary MR, *n* (%)	68 (100.0)	111 (76.5)^*^	28 (82.3)^*^	<0.001
Presence of MAC, *n* (%)	6 (8.7)	19 (5.8)	6 (17.6)^#^	0.035
**Post-KT echocardiogram**				
LVEDD, mm	48.7 ± 5.6	48.9 ± 5.8	52.0 ± 5.8	0.011
LVESD, mm	30.3 ± 5.6	30.4 ± 5.1	33.5 ± 5.7	0.006
LVEF, %	65.4 ± 7.6	65.8 ± 7.8	58.4 ± 9.6	<0.001
LV mass index, g/m^2^	108.3 ± 34.4	111.7 ± 33.5	125.6 ± 33.9	0.045
LA volume index, ml/m^2^	36.8 ± 14.4	35.5 ± 16.3	45.0 ± 17.8	0.006
E/e′	11.6 ± 5.1	11.5 ± 4.8	16.6 ± 7.2^*^^#^	<0.001
PASP, mmHg	30.3 ± 11.6	29.5 ± 7.9	42.7 ± 13.4^*^^#^	0.003
Degree of MR, *n* (%)				<0.001
No/trivial	51 (73.9)	190 (58.1)^*^	0 (0.0)^*^^#^	
Mild	17 (24.6)	132 (40.4)^*^	24 (70.6)^*^^#^	
Moderate	1 (1.4)	5 (1.5)	10 (29.4)^*^^#^	
Severe	0 (0.0)	0 (0.0)	0 (0.0)	
**Changes after KT**				
Δ LVEDD, mm	−1.8 ± 9.6	1.1 ± 9.4	0.9 ± 5.8	0.082
Δ LVESD, mm	−8.1 ± 8.8	−3.7 ± 5.4^*^	0.9 ± 6.5^*^	<0.001
Δ LVEF, %	8.3 ± 13.6	3.5 ± 8.4	−3.3 ± 10.6^*^	<0.001
Δ E/e′	−4.4 ± 6.5	−1.1 ± 5.4^*^	4.2 ± 5.3^*^^#^	<0001
Δ LV mass index, g/m^2^	−42.8 ± 53.4	−12.5 ± 46.6	7.4 ± 42.7^*^^#^	<0.001
Δ LA volume index, ml/m^2^	−15.7 ± 24.7	−4.7 ± 13.8	1.0 ± 17.7^*^	<0.001

* *P < 0.05 compared with the MR regression group*.

# *P < 0.05 compared with the MR unchanged group*.

*MR, mitral regurgitation; LVEDD, left ventricular end-diastolic diameter; LVESD, lefMRt ventricular end-systolic diameter; LVEF, left ventricular ejection fraction; LV, left ventricle; LA, left atrium; E/e′, ratio of early diastolic mitral velocity to early diastolic mitral annular velocity; PASP, pulmonary arterial systolic pressure; MAC, mitral annular calcification*.

**Figure 2:**
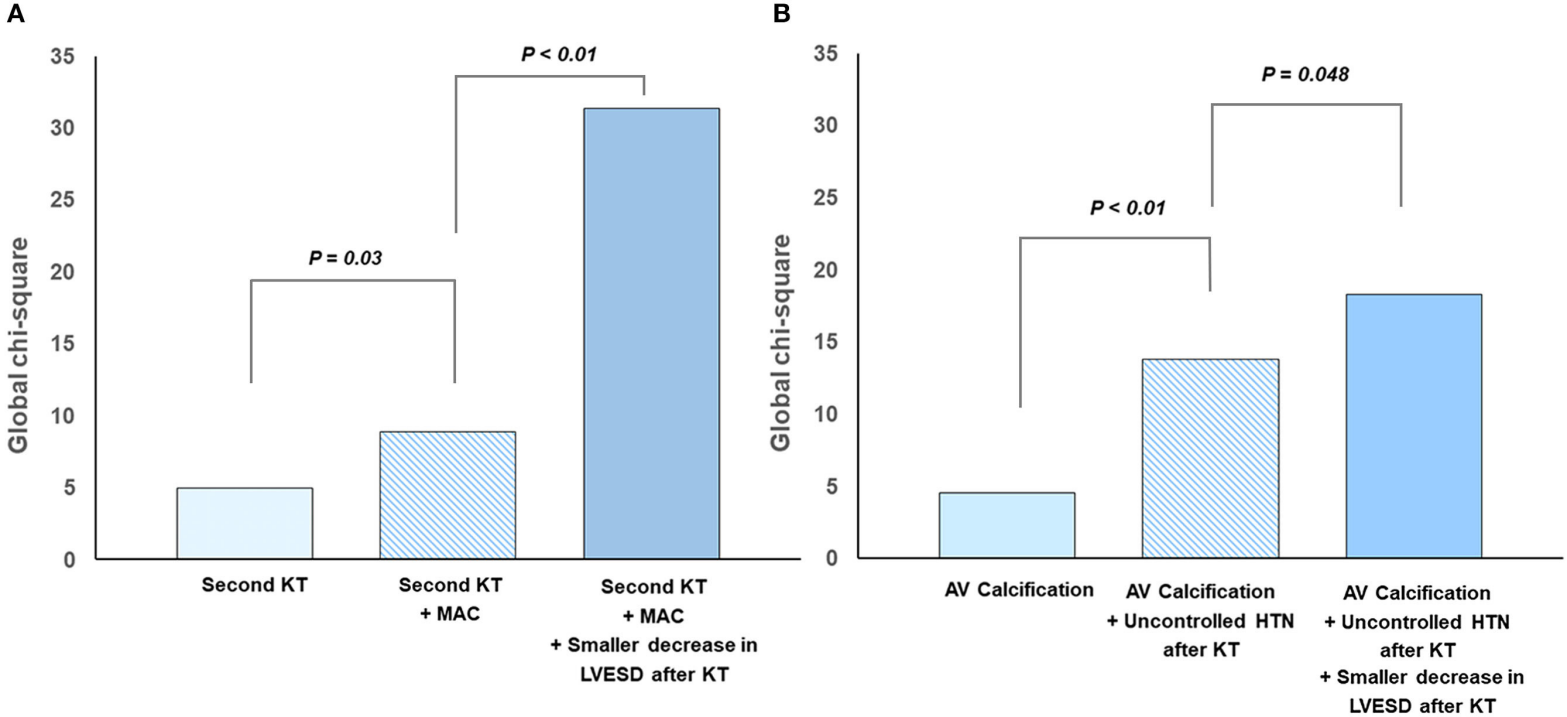
Predictors for progression of left-sided valvular regurgitation after kidney transplantation: **(A)** mitral regurgitation and **(B)** aortic regurgitation.

Univariate linear regression analysis revealed that second KT, MAC before KT, renal dysfunction after KT, and a smaller decrease in LV end systolic dimension (LVESD) were associated with MR progression. After multivariable adjustment, second KT, baseline MAC, and smaller decrease in LVESD after KT were independent predictors for MR progression after KT (Table 3). Factors related to progression of MR showed statistically meaningful predictive values in a stepwise manner (Figure 2A).

**Table 3:** Factors associated with progression of mitral regurgitation after KT.

	**Univariate analysis**			**Multivariate analysis**		
	***B***	***t***	***p*** **-value**	***B***	***t***	***p*** **-value**
Age	0.002	1.82	0.070	0.001	1.29	0.198
Female sex	0.016	0.61	0.541			
Hypertension	−0.041	−0.95	0.345			
Diabetes mellitus	0.038	1.41	0.158			
Dyslipidemia	−0.052	−1.56	0.119			
Atrial fibrillation	−0.084	−1.41	0.158			
CAD	0.060	1.52	0.129			
Heart failure	−0.031	−0.67	0.501			
HD duration	−0.032	−0.59	0.558			
Second KT	0.151	3.07	0.002	0.114	2.31	0.022
New-onset HTN	0.027	0.43	0.669			
Persistent HTN	0.036	1.21	0.228			
New-onset DM	−0.034	−0.90	0.367			
Renal dysfunction	0.081	2.47	0.014	0.062	1.19	0.057
Graft failure	0.046	1.14	0.255			
Secondary MR	−0.068	−1.07	0.286			
Presence of MAC	0.123	2.46	0.014	0.110	2.20	0.028
Δ LVEDD	0.008	0.16	0.876			
Δ LVESD	0.009	4.57	<0.001	0.008	4.15	<0.001
Δ LVEF	−0.006	−4.21	<0.001			
Δ LV mass index	0.001	2.79	0.006			
Δ LA volume index	0.002	2.43	0.016			

*CAD, coronary artery disease; HD, hemodialysis; KT, kidney transplantation; HTN, hypertension; DM, diabetes mellitus; MR, mitral regurgitation; MAC, mitral annular calcification; LVEDD, left ventricular end-diastolic diameter; LVESD, left ventricular end-systolic diameter; LVEF, left ventricular ejection fraction; LV, left ventricle; LA, left atrium*.

These findings indicate that MR tended to show regression after KT for controlling volume overload. In contrast, MR progression can occur after KT if the volume overload is relatively low before KT or if valve degeneration represented by MAC is present before KT. Furthermore, progression of MR was related to subsequent elevation of LV filling pressure after KT.

### Factors Associated With AR Progression

Table 4 shows the baseline clinical and renal characteristics of the three groups categorized by AR change after KT. Patients who showed AR progression experienced persistent hypertension, defined as systolic blood pressure ≥140 mmHg and diastolic blood pressure ≥90 mmHg after KT among those with hypertension. In echocardiographic findings, patients who showed AR progression showed larger LV and LA size after KT compared with the other groups. Similar to the results from the analysis of MR, smaller reductions in LV and LA sizes after KT, no improvement in LVEF, and an increase in E/e′ were observed in the AR progression group (Table 5).

**Table 4:** Comparison of clinical and renal characteristics in the three groups according to change in aortic regurgitation.

	**AR regression** **(***n*** = 38)**	**AR unchanged** **(***n*** = 355)**	**AR progression** **(***n*** =37)**	***p*** **-value**
**Clinical characteristics**				
Age, years	53.2 ± 12.9	49.4 ± 12.8	53.7 ± 9.8	0.044
Male sex, *n* (%)	11 (28.9)	198 (55.8)^*^	15 (40.5)	0.002
Hypertension, *n* (%)	32 (84.2)	320 (90.4)	34 (91.9)	0.445
Diabetes mellitus, *n* (%)	14 (36.8)	124 (35.0)	16 (43.2)	0.607
Dyslipidemia, *n* (%)	6 (15.8)	71 (20.1)	4 (10.8)	0.345
Atrial fibrillation, *n* (%)	5 (13.2)	16 (4.5)	1 (2.7)	0.056
CAD, *n* (%)	6 (15.8)	43 (12.1)	4 (10.8)	0.775
Heart failure, *n* (%)	8 (21.1)	28 (7.9)^*^	3 (8.1)	0.027
**Renal characteristics**				
Dialysis, *n* (%)	38 (100.0)	355 (100.0)	37 (100.0)	1.000
Hemodialysis, *n* (%)	35 (92.1)	305 (85.9)	35 (94.6)	0.473
Peritoneal dialysis, *n* (%)	3 (7.9)	50 (14.1)	2 (5.4)	0.519
HD duration, months	62.9 ± 64.6	64.3 ± 66.6	55.0 ± 54.9	0.733
Second KT, *n* (%)	4 (10.5)	24 (6.8)	4 (10.8)	0.503
**Post-KT comorbidities**, ***n*** **(%)**				
New-onset HTN	3 (7.9)	15 (4.2)	1 (2.7)	0.504
Persistent HTN	9 (23.7)	103 (29.0)	21 (56.8)^*^^#^	0.001
New-onset DM	3 (7.9)	54 (15.3)	3 (8.1)	0.258
Renal dysfunction, *n* (%)	4 (10.5)	69 (19.4)	10 (27.0)	0.192
Graft failure, *n* (%)	10 (26.3)	33 (9.3)^*^	7 (18.9)	0.003
**Post-KT medications**, ***n*** **(%)**				
RAAS blocker	2 (5.2)	91 (30.3)^*^	3 (8.1)^#^	<0.001
Beta blocker	16 (43.2)	149 (49.5)	17 (45.9)	0.731
CCB	16 (34.0)	267 (33.7)	20 (37.0)	0.630
Diuretics	4 (10.8)	32 (10.8)	1 (2.7)	0.296
Statin	0 (0)	110 (36.9)^*^	4 (11.1)^*^^#^	<0.001

* *P < 0.05 compared with the AR regression group*.

# *P < 0.05 compared with the AR unchanged group*.

*AR, aortic regurgitation; CAD, coronary artery disease; HD, hemodialysis; KT, kidney transplantation; HTN, hypertension; DM, diabetes mellitus; RAAS, renin-angiotensin-aldosterone system; CCB, calcium channel blocker*.

**Table 5:** Comparison of echocardiographic characteristics in the three groups according to change in aortic regurgitation.

	**AR regression** **(***n*** = 38)**	**AR unchanged** **(***n*** = 355)**	**AR progression** **(***n*** =37)**	***p*** **-value**
**Pre-KT echocardiogram**				
LVEDD, mm	55.6 ± 8.0	52.6 ± 6.1^*^	53.7 ± 4.5	0.018
LVESD, mm	36.4 ± 10.2	34.6 ± 6.5	32.9 ± 5.4	0.095
LVEF, %	58.7 ± 13.5	61.5 ± 9.9	63.4 ± 8.8	0.141
LV mass index, g/m^2^	139.7 ± 55.8	127.0 ± 39.0	128.6 ± 34.4	0.195
LA volume index, ml/m^2^	54.1 ± 29.8	38.5 ± 14.8^*^	45.3 ± 19.2	<0.001
E/e′	14.9 ± 6.9	12.7 ± 5.7	12.6 ± 5.1	0.113
PASP, mmHg	36.6 ± 11.2	30.0 ± 10.3	29.8 ± 9.1	0.006
Degree of AR, *n* (%)				<0.001
No/trivial	0 (0)	296 (83.4)^*^	35 (94.6)^*^	
Mild	34 (89.5)	47 (13.2)^*^	2 (5.4)^*^^#^	
Moderate	4 (10.5)	10 (2.8)^*^	0 (0)^*^	
Severe	0 (0)	2 (0.6)	0 (0)	
AV calcification, *n* (%)	4 (10.5)	33 (9.3)	8 (21.6)^#^	0.018
**Post-KT echocardiogram**				
LVEDD, mm	48.7 ± 6.7	48.8 ± 5.8	51.9 ± 4.7^*^^#^	0.008
LVESD. Mm	28.7 ± 5.5	30.7 ± 5.3	31.8 ± 5.1^*^	0.033
LVEF, %	66.5 ± 7.4	65.2 ± 8.2	63.1 ± 7.9	0.185
LV mass index, g/m^2^	112.9 ± 34.9	111.8 ± 34.0	116.7 ± 31.7	0.706
LA volume index, ml/m^2^	43.2 ± 18.6	35.0 ± 15.4^*^	44.0 ± 19.5^#^	<0.001
E/e′	12.0 ± 6.5	11.6 ± 4.7	14.7 ± 7.1	0.005
PASP, mmHg	33.1 ± 12.5	28.7 ± 9.9	31.0 ± 9.2	0.083
Degree of AR, *n* (%)				<0.001
No/trivial	34 (89.5)	296 (83.4)	0 (0)^*^^#^	
Mild	4 (10.5)	51 (14.4)	34 (91.9)^*^^#^	
Moderate	0 (0)	8 (2.2)	3 (8.1)^*^^#^	
Severe	0 (0)	0 (0)	0 (0)	
**Changes after KT**				
Δ LVEDD, mm	−4.5 ± 9.1	1.4 ± 9.7^*^	−1.1 ± 6.4	0.001
Δ LVESD, mm	−7.5 ± 9.8	−4.0 ± 6.0^*^	−1.2 ± 5.7^*^^#^	<0.001
Δ LVEF, %)	7.3 ± 13.6	3.7 ± 9.4	−0.2 ± 9.1^*^^#^	0.006
Δ E/e′	−3.6 ± 6.9	−1.3 ± 5.7	2.5 ± 5.6^*^	<0.001
Δ LV mass index, g/m^2^	−30.4 ± 55.6	−14.3 ± 48.6	−11.2 ± 45.3	0.193
Δ LA volume index, ml/m^2^	−13.4 ± 28.9	−5.7 ± 14.6^*^	0.2 ± 18.1^*^	0.004

* *P < 0.05 compared with the AR regression group*.

# *P < 0.05 compared with the AR unchanged group*.

*AR, aortic regurgitation; LVEDD, left ventricular end-diastolic diameter; LVESD, left ventricular end-systolic diameter; LVEF, left ventricular ejection fraction; LV, left ventricle; LA, left atrium; E/e′, ratio of early diastolic mitral velocity to early diastolic mitral annular velocity; PASP; pulmonary arterial systolic pressure; AV, aortic valve*.

Univariate linear regression analysis revealed that presence of aortic valve calcification before KT, persistent HTN after KT, and a smaller decrease in LVESD after KT were associated with AR progression. All the above factors were independent predictors for AR progression after KT in multivariate analysis (Table 6). Figure 2B shows additive predictive values for AR progression after KT in a stepwise manner. These findings suggest that preexisting aortic valve degeneration accompanying unresolved afterload as well as less volume control after KT might affect AR progression.

**Table 6:** Factors associated with progression of aortic regurgitation after KT.

	**Univariate analysis**			**Multivariate analysis**		
	***B***	***t***	***p*** **-value**	***B***	***t***	***p*** **-value**
Age	0.002	1.80	0.073	−0.002	−0.63	0.533
Female sex	0.040	1.47	0.142			
Hypertension	0.018	0.41	0.686			
Diabetes mellitus	0.028	0.97	0.331			
Dyslipidemia	−0.045	−1.13	0.190			
Atrial fibrillation	−0.043	−0.70	0.485			
CAD	−0.012	−0.30	0.766			
Heart failure	−0.010	−0.21	0.832			
HD duration	−0.042	−0.78	0.435			
New-onset HTN	−0.035	−0.53	0.594			
Second KT	0.042	0.82	0.415			
Persistent HTN	0.063	2.07	0.039	0.200	2.49	0.014
New-onset DM	−0.042	−1.08	0.282			
Renal dysfunction	0.043	1.25	0.214			
Graft failure	0.061	1.45	0.149			
AV calcification	0.330	2.66	0.009	0.274	0.12	0.029
Δ LVEDD	−0.002	−1.11	0.269			
Δ LVESD	0.006	2.73	0.007	0.010	2.01	0.046
Δ LVEF	−0.003	−2.46	0.014			
Δ LV mass index	0.027	0.52	0.603			
Δ LA volume index	0.002	2.21	0.028			

*CAD, coronary artery disease; HD, hemodialysis; KT, kidney transplantation; HTN, hypertension; DM, diabetes mellitus; AV, aortic valve; LVEDD, left ventricular end-diastolic diameter; LVESD, left ventricular end-systolic diameter; LVEF, left ventricular ejection fraction; LV, left ventricle; LA, left atrium*.

## Discussion

The principal findings in the present multicenter study are as follows. (1) Left-side valve regurgitation is common in patients with ESRD undergoing KT, and MR is more common than AR. (2) After KT, MR and AR regress or do not change in most patients but progress in some patients. (3) Patients who receive second KT and who have MAC and a smaller decrease in LVESD after KT showed MR progression after KT. (4) Patients who had aortic valve calcification, persistent HTN after KT, and a smaller decrease in LVESD after KT showed AR progression. The present study suggests that pre-existing valve degeneration before KT, smaller volume changes, and specific conditions before or after KT affect the progression of regurgitation on each valve. As expected, MR was affected more highly by volume factor, and AR was affected most by afterload, such as persistent HTN after KT. Therefore, it is necessary to perform echocardiographic surveillance and risk factor control after KT according to individual characteristics before and after KT and presence of valve calcification.

### Left-Side Valve Disease in Patients With ESRD

Patients with chronic kidney disease or ESRD show a high prevalence of left-side valve disease due to not only degeneration of the valve itself, but also chamber dilatation or dysfunction related with increased hemodynamic load and loss of LV contractility (8, 17). Previous studies demonstrated higher prevalence of premature aortic valve calcification and consequent aortic stenosis, as well as mitral annular calcification and functional mitral stenosis (18–21). In particular, increased calcium x phosphate product and long-term hemodialysis are associated with valve calcification (8, 17). Also, increased cardiac output caused by anemia and arteriovenous fistula and hypertension increase mechanical stress in the valve leaflets and modulate premature valve calcification (8, 17). In stenotic valve disease, primary degeneration is the main mechanism, whereas regurgitation is caused mainly by secondary causes. Therefore, because of preload and afterload reduction after KT and additional improvement of renin-angiotensin-aldosterone system (RAAS) activation, regression of valve regurgitation is expected after KT. If the patient does not show any specific clinical features after KT, regular echocardiographic follow-up is not performed; valve regurgitation sometimes progresses, and timely treatment can be delayed.

### Natural Course of MR and AR in Patients Undergoing KT

Several previous studies have focused on valve disease in patients undergoing KT. A serial echocardiographic follow-up study in 95 patients undergoing KT reported no interval change in average MR fraction and volume after KT (22). However, this study was limited in that it did not provide information on patient characteristics or changes in chamber size and function. Another study conducted at a single center with 180 patients undergoing KT demonstrated that grade 2 MR decreased from 11% on pre-KT echocardiogram to 2% at 12 months after KT (23). Interestingly, valve calcification was detected preoperatively in 21.5% of the study population but was detected in 25.8% of the population at 6 months after KT and in 35.5% of the population 12 months after KT (23). Incidence of calcified valve was more common in patients with diabetes than in those without it (23). These results suggest that, even if hemodynamic load is resolved by KT, valve degeneration and calcification can have initiated and can proceed. It also suggests that risk factor modulation and echocardiographic follow-up are needed even after KT in patients who have calcification on the valve or surrounding annular structure. In this context, the present study showed on pre-KT echocardiogram that MR and AR can progress even after KT in patients with MAC or aortic valve calcification through a multicenter analysis. In addition, we found some differential risk factors between MR progression and AR progression after KT.

### Risk Factors for Progression of MR and AR After KT

Smaller LVESD decrease after KT was related to progression of not only MR, but also AR in this study. Therefore, a smaller volume decrease after KT was a pre-condition of progression of left-side valve regurgitation. These results indicate the need for follow-up TTE for surveillance of chamber size and course of functional regurgitation after KT, especially for patients who have predisposing factors. Furthermore, prolonged optimal medical treatment might be necessary after KT, especially for patients with risk factors of progression. In our study, there might have been insufficient usage of RAAS inhibitor in patients with progression of AR compared with that in the unchanged AR group. Since RASS inhibitor can promote reverse LV remodeling after KT through both afterload reduction and inhibition of myocardial fibrosis, active use of RASS inhibitor after KT seems to be helpful for hemodynamic control.

MAC or aortic valve calcification shares biological links with atherosclerosis and is very common in patients with ESRD (18). Patients with valve or peri-valvular calcification in ESRD were more likely to have dyslipidemia and a broken mineral-bone axis, and this condition might not recover fully after KT (16). These conditions were thought to be due to valve dysfunction accompanied by valve calcification that could progress after KT. This might be related to greater use of statins in patients who showed progression of AR after KT.

After KT, hypertension remains widespread, with 56–93% of recipients consistently having a systolic blood pressure >140 mmHg. Multiple factors can lead to hypertension, including donor and recipient characteristics, immunosuppressive medications, and allograft function (17). Long-term exposure to high blood pressure is a strong and potentially modifiable risk factor for aortic stenosis and regurgitation (19). Our study also revealed persistent HTN after KT as a predictor of AR progression. This suggests that unresolved afterload and preload affect AR progression; therefore, strict anti-hypertensive treatment is important for preventing progression of AR after KT.

### Study Limitations

First, this was a retrospective study and a significant number of patients were excluded because there was no echocardiographic follow-up within a specific time period after KT. Moreover, post-KT echocardiography was not performed according to the pre-specified period. However, we attempted to overcome these shortcomings by including a large number of patients from multiple centers who had undergone follow-up echocardiography between 6 and 36 months after KT.

Second, in this KT cohort, there were many cases of no or mild valve regurgitation before KT. If there was significant or severe valve regurgitation, it is possible that KT was postponed or not performed, and volume control by dialysis was attempted. Accordingly, there were many patients whose MR and AR did not change after KT. There was a limitation of the population in evaluating the dynamic change of AR and MR. Therefore, it is possible that we selected only relatively healthy ESRD patients. Nevertheless, we believe that we identified patient characteristics that reflect real clinical practice and the natural course of MR and AR.

Third, although we aimed to find factors that could influence progression of MR and AR, it is possible that some factors that were not investigated or that undetected confounding factors had an influence on the results.

## Conclusions

Among patients undergoing KT, MR, and AR can progress in those patients with certain characteristics. Risk factors for progression of MR after KT include a second KT, MAC and a smaller decrease in LVESD after KT. Risk factors for progression of AR include valve calcification, persistent hypertension and a smaller decrease in LVESD after KT. Further echocardiographic surveillance and risk factor management after KT are warranted in these patients.

## Data Availability Statement

The original contributions presented in the study are included in the article/Supplementary Material, further inquiries can be directed to the corresponding author.

## Ethics Statement

The studies involving human participants were reviewed and approved by Institutional Review Board of the Yonsei University Health System (2021-0994-001). Written informed consent for participation was not required for this study in accordance with the national legislation and the institutional requirements.

## Author Contributions

MK reviewed the literature and wrote the first draft of the manuscript. CS supervised this paper, coordinated the multidisciplinary discussion, and critically reviewed the manuscript. DK provided the data of the Samsung medical center and reviewed the manuscript. JL, D-YK, JS, IC, KH, G-RH, and J-WH reviewed the manuscript, provided comments, and suggested modifications to the manuscript. All authors contributed to the article and approved the submitted version.

## Conflict of Interest

The authors declare that the research was conducted in the absence of any commercial or financial relationships that could be construed as a potential conflict of interest.

## Publisher's Note

All claims expressed in this article are solely those of the authors and do not necessarily represent those of their affiliated organizations, or those of the publisher, the editors and the reviewers. Any product that may be evaluated in this article, or claim that may be made by its manufacturer, is not guaranteed or endorsed by the publisher.
