# Supplementary material for: Mitral and Aortic Regurgitation in Patients Undergoing Kidney Transplantation: The Natural Course and Factors Associated With Progression
Source: Front Cardiovasc Med. 2022 Jan 27;9:809707. doi: 10.3389/fcvm.2022.809707 (PMC8829463; doi:10.3389/fcvm.2022.809707)
Supplement: Supplementary file 1 [file Data_Sheet_1.docx]

**Supplementary Table 1. Factors associated with pre-KT mitral regurgitation grade**

|  | **Univariate analysis** | | | **Multivariate analysis** | | |
| --- | --- | --- | --- | --- | --- | --- |
|  | **B** | **t** | **p-value** | **B** | **t** | **p-value** |
| Age | 0.145 | 3.03 | 0.003 | 0.003 | 1.264 | 0.207 |
| Female sex | 0.176 | 3.69 | <0.001 | 0.136 | 2.263 | 0.024 |
| Hypertension | -0.062 | -1.28 | 0.203 |  |  |  |
| Diabetes mellitus | 0.032 | 0.65 | 0.513 |  |  |  |
| Dyslipidemia | -0.053 | -1.11 | 0.269 |  |  |  |
| Atrial fibrillation | 0.215 | 4.54 | <0.001 | 0.071 | 0.501 | 0.617 |
| CAD | 0.119 | 2.47 | 0.014 | 0.077 | 0.773 | 0.440 |
| Heart failure | 0.247 | 5.27 | <0.001 | 0.316 | 2.794 | 0.005 |
| HD duration | -0.078 | -1.15 | 0.149 |  |  |  |
| Second KT | -0.051 | -1.07 | 0.287 |  |  |  |
| Presence of MAC | 0.048 | 0.99 | 0.323 |  |  |  |
| Pre-LVEDD | 0.399 | 8.81 | <0.001 |  |  |  |
| Pre-LVESD | 0.274 | 5.77 | <0.001 | 0.005 | 0.943 | 0.346 |
| Pre-LVEF | -0.319 | -6.80 | <0.001 |  |  |  |
| Pre-LV mass index | 0.231 | 4.73 | <0.001 |  |  |  |
| Pre-LA volume index | 0.490 | 11.45 | <0.001 | 0.016 | 8.317 | <0.001 |
| Pre E/e’ | 0.249 | 5.05 | <0.001 |  |  |  |

CAD, coronary artery disease; HD, hemodialysis; KT, kidney transplantation; MAC, mitral annular calcification; LVEDD, left ventricular end-diastolic diameter; LVESD, left ventricular end-systolic diameter; LVEF, left ventricular ejection fraction; LV, left ventricle; LA, left atrium; E/e’, ratio of early diastolic mitral velocity to early diastolic mitral annular velocity

**Supplementary Table 2. Factors associated with pre-KT aortic regurgitation grade**

|  | **Univariate analysis** | | | **Multivariate analysis** | | |
| --- | --- | --- | --- | --- | --- | --- |
|  | **B** | **t** | **p-value** | **B** | **t** | **p-value** |
| Age | 0.209 | 4.42 | <0.001 | 0.008 | 3.799 | <0.001 |
| Female sex | 0.086 | 1.78 | 0.075 | 0.049 | 0.910 | 0.364 |
| Hypertension | 0.010 | 0.22 | 0.829 |  |  |  |
| Diabetes mellitus | 0.009 | 0,19 | 0.853 |  |  |  |
| Dyslipidemia | -0.067 | -1.39 | 0.165 |  |  |  |
| Atrial fibrillation | 0.117 | 2.44 | 0.015 | 0.049 | 0.388 | 0.698 |
| CAD | 0.060 | 1.23 | 0.218 |  |  |  |
| Heart failure | 0.186 | 3.92 | <0.001 | 0.292 | 2.988 | 0.003 |
| HD duration | -0.021 | -0.40 | 0.691 |  |  |  |
| Second KT | 0.038 | 0.78 | 0.436 |  |  |  |
| AV calcification | -0.257 | -1.423 | 0.157 |  |  |  |
| Pre-LVEDD | 0.157 | 3.22 | 0.001 |  |  |  |
| Pre-LVESD | 0.104 | 2.11 | 0.036 | -0.002 | -0.380 | 0.704 |
| Pre-LVEF | -0.128 | -2.61 | 0.009 |  |  |  |
| Pre-LV mass index | 0.120 | 2.40 | 0.017 |  |  |  |
| Pre-LA volume index | 0.162 | 3.34 | 0.001 | 0.003 | 1.464 | 0.144 |
| Pre-E/e’ | 0.094 | 1.85 | 0.066 |  |  |  |

CAD, coronary artery disease; HD, hemodialysis; KT, kidney transplantation; AV, aortic valve; LVEDD, left ventricular end-diastolic diameter; LVESD, left ventricular end-systolic diameter; LVEF, left ventricular ejection fraction; LV, left ventricle; LA, left atrium; E/e’, ratio of early diastolic mitral velocity to early diastolic mitral annular velocity
